# 

**Published:** 1914-03

**Authors:** 


### CONTENTS OF VOLUME XXXII.

---

*Page*

Diseases of the Lungs associated with the Presence of Friedländer's Bacillus. By J. MICHELL CLARKE, M.A., M.D., LL.D., F.R.C.P. 1

Some Unusual Conditions found in Operating for the Radical Cure of Hernia. By CHARLES A. MORTON, F.R.C.S. .. .. .. 12

On Digital Percussion and the Cardiac Sign in Carcinoma. By W. GORDON, M.A., M.D. Cantab., F.R.C.P. .. .. .. .. 19

The Simulation of Aortic Aneurysm by some other aortic and Cardiac Diseases. By CAREY COOMBS, M.D., F.R.C.P. Lond. .. 26

Fibroid Uterus. By W. H. C. NEWNHAM, M.A., M.B. Cantab. .. 37

Hyperpiesis and Arterio-Sclerosis. By C. W. J. BRASHER, M.D. .. 41

Notes on the Electrical Reactions in Facial Paralysis, especially in reference to the Prognosis in Post-Operative Cases. By JOHN P. I. HARTY, B.A., M.B., B.Ch., F.R.C.S. .. .. 55

The Secondary Infections of Pulmonary Tuberculosis and their Treatment by Vaccines. By S. H. HABERSHON, M.D. Cantab., F.R.C.P. .. .. .. .. .. .. .. .. 97

Mixed Infections in Pulmonary Tuberculosis, and some General Observations on Treatment with Tuberculin. By NOEL BARDSWELL, M.D. Edin., M.R.C.P. Lond. and Edin., F.R.S.E. .. 125

Note on the Treatment of Laryngeal Tuberculosis by Tuberculin. By P. WATSON-WILLIAMS, M.D. Lond. .. .. .. .. 136

Auto-Plastic Intra-Medullary Bone Pegging as a Method of Operative Treatment for Fractures. By C. FERRIER WALTERS, F.R.C.S... 139

On the Occurrence of Tachycardia in Association with Parenchymatous Goitre. By F. H. EDGEWORTH, M.D. Cantab., D.Sc. Lond. .. .. .. .. .. .. .. .. 144

Notes on a Case of Lymphatic Leukæmia in a Child Aged Three Years. By EDWARD CECIL WILLIAMS, B.A., M.B. Cantab. .. 147

Local Analgesia in Surgical Operations. By A. L. FLEMMING, M.B., Ch.B. Bristol .. .. .. .. .. .. .. .. 193

CONTENTS.

| | Page |
|---|---|
| A Case of Richter's Strangulated Hernia. By C. A. Moore, M.S. Lond., Ch.M. Bristol, F.R.C.S. | 198 |
| The Radiographic Centroscope and the Radiographic Episcope. By William Cotton, M.D. Edin. | 202 |
| Spiritual Healing: A Review | 214 |
| The Evolution of Gynæcology. By W. H. C. Newnham, M.A., M.B. Cantab. | 257 |
| Notes on Some Bristol Medical Societies. By G. Munro Smith, M.D. Bristol | 268 |
| The Clinical Manifestations and Treatment of Coliuria in Children. By R. G. Gordon, M.D., B.Sc., M.R.C.P. Edin. | 286 |
| Some Impressions of the 1914 British Pharmacopœia. By Oliver C. M. Davis, D.Sc. | 292 |
| Progress of the Medical Sciences | 63, 151, 217, 297 |
| Reviews of Books | 72, 164, 229, 305 |
| Editorial Notes | 177, 239, 315 |
| Therapeutic Notes | 88, 181, 249 |
| The Library of the Bristol Medico-Chirurgical Society | 91, 184, 252, 316 |
| Meetings of Societies | 93, 187, 317 |
| Obituary | 189, 253 |
| Local Medical Notes | 96, 192, 256, 320 |

